# Genome Sequence of the Uncommon *Streptococcus pyogenes* M/*emm*66 Strain STAB13021, Isolated from Clonal Clustered Cases in French Brittany

**DOI:** 10.1128/genomeA.00689-16

**Published:** 2016-07-21

**Authors:** Alexandra Meygret, Pascal Vincent, Séverine Moullec, Jessica Nacazume, Yahia Adnani, Dominique Lavenier, Samer Kayal, Ahmad Faili

**Affiliations:** a CHU Rennes-Service de Bactériologie et Hygiène Hospitalière, Rennes, France; b CNRS, IGDR-UMR 6290, Team GeRMO, Rennes, France; c National Institute for Research in Computer Science and Control, IRISA/GenScale, Team Bioinformatique, Campus de Beaulieu, Rennes, France

## Abstract

Here, we announce the complete annotated genome sequence of the invasive *Streptococcus pyogenes* strain M/*emm*66, isolated in 2013 from a subcutaneous abscess in new clustered cases in French Brittany.

## GENOME ANNOUNCEMENT

Group A *Streptococcus pyogenes* (GAS) is a Gram-positive human pathogen that causes a broad range of invasive and noninvasive diseases (1, 2). Outbreak of infections due to GAS have long been known to be a major cause of human morbidity and mortality (3); however, the molecular basis of pathogen emergence and differences in strain virulence are still poorly understood.

Since 2009, we have been conducting a systematic survey of all GAS isolates in our geographical area based on *emm*-genotyping (4, 5) and have observed a clonal emergence (*n* = 13 isolates with the same pulsed field electrophoresis) of an unusual *emm*66 genotype (6–8). We sequenced and annotated the whole genome of an invasive GAS-M/*emm66* strain isolated in 2013 from a subcutaneous abscess, henceforth named STAB13021.

Bacterial growth and DNA extraction were performed as previously described (9, 10). Genomic DNA was sequenced using HiSeq 2000 technology (Illumina, Inc., San Diego, CA, USA), and the paired-end library was built using the MGX facility of the CNRS in Montpellier, France. There is a total of 13,785,510 high-quality reads giving an average 720-fold coverage of the genome, which was assembled using CLC Genomics Workbench version 6 software (http://www.clcbio.com). The resulting assembly consisted of 31 contigs, which were oriented on the basis of available sequences of GAS. After reassembling, 23 gaps persisted, which were filled by PCR followed by Sanger sequencing. Genome annotation was performed in parallel using the RAST server (11) and the NCBI PGAP (http://ncbi.nlm.nih.gov/genome/annotation_prok). Prophages were identified using the PHAge search tool (PHAST) (12).

Finally, strain STAB13021 harbored a single circular genome of 1,810,577 bp, with a G+C content of 38.35%. We identified 1,663 coding sequences (CDSs), 53 tRNAs genes, 18 rRNAs genes, two intact integrated prophages (38.6 kb, 61 CDSs, G+C content of 38.30% and 55.6 kb, 74 CDSs, G+C content of 38.96%), and one possible incomplete phage (inserted in the noncoding region) between the *mutS* and *mutL* genes (13.5 kb, 21 CDSs, G+C content of 36.5%). The web-based tool CRISPRFinder identified one candidate clustered regularly interspaced short palindromic repeat (CRISPR) region (13). The multilocus sequence type (14) was determined to be ST44.

Among known virulence factors the pyrogenic exotoxin *speB* gene and the superantigen *speG* and s*me*Z genes were identified in the chromosome, while mitogenic factors (*mf2* and *mf3*), streptodornase (*sda*), and streptokinase A (*skA*) were not found. Interestingly, further analysis identified a single mutation in *ropB* coding for the stand-alone regulator RopB, resulting in a premature stop codon. This null allele may be associated with an absence of SpeB activity or, as described recently, may incur an overall fitness cost for GAS, preventing its fixation in the population (15).

The annotated sequence of this rare GAS *emm66* genotype and newly emerged clone provides a genetic basis for comparison in order to enhance the understanding of clone emergence and hopefully control measures and vaccine design strategies.

### Nucleotide sequence accession number.

The complete genome sequence of strain STAB13021 has been deposited in the NCBI GenBank under the accession number CP014278, as part of Bioproject PRJNA310843.
